# Case Report: Glomerular microangiopathy and reversible renal toxicity during prolonged bevacizumab therapy in ovarian cancer

**DOI:** 10.3389/fonc.2026.1752830

**Published:** 2026-01-23

**Authors:** Jungheon Kwon, Misun Choe, Jin Hyuk Paek, Woo Yeong Park, Kyubok Jin, Seungyeup Han, Chi-Heum Cho, Yaerim Kim

**Affiliations:** 1 Division of Nephrology, Department of Internal Medicine, Gyeongsan Joongang Hospital, Daegu, Republic of Korea; 2 Department of Pathology, Keimyung University School of Medicine, Daegu, Republic of Korea; 3 Division of Nephrology, Department of Internal Medicine, Keimyung University School of Medicine, Daegu, Republic of Korea; 4 Department of Obstetrics and Gynecology, Keimyung University School of Medicine, Daegu, Republic of Korea

**Keywords:** bevacizumab, glomerular microangiopathy, nephrotic syndrome, proteinuria, vascular-endothelial growth factor

## Abstract

**Background:**

Bevacizumab, a VEGF-A inhibitor widely used in ovarian cancer, is associated with kidney adverse effects ranging from mild proteinuria to thrombotic microangiopathy (TMA). However, the longitudinal course, reversibility, and spectrum of kidney-limited injury remain under-characterized.

**Case report:**

We describe three patients with ovarian cancer who developed proteinuria during prolonged bevacizumab therapy, including one with biopsy-proven kidney-limited glomerular microangiopathy. Proteinuria emerged after 6 to 12 months of exposure in all cases. Kidney function remained preserved throughout treatment. All three patients discontinued bevacizumab, after which proteinuria consistently improved. Two patients experienced complete resolution within 3 to 6 months, and one showed marked reduction to dipstick 1 plus by three months. Kidney biopsy in one case revealed double-contour basement membrane changes and subendothelial electron-dense deposits, confirming kidney-limited TMA without systemic microangiopathic findings.

**Conclusion:**

These cases illustrate the characteristic time course of bevacizumab-induced kidney toxicity, highlight the reversibility of proteinuria following drug discontinuation, and demonstrate that bevacizumab can induce kidney-limited TMA in the absence of systemic microangiopathic features. Multidisciplinary management is essential to balance oncologic benefit with kidney safety.

## Highlights

- Bevacizumab can cause proteinuria and kidney-limited thrombotic microangiopathy even without systemic features of TMA.
- Proteinuria typically develops within the first year of therapy and in most cases improves after bevacizumab discontinuation
- Kidney biopsy can guide management and avoid unnecessary systemic treatment when microangiopathy is kidney-limited.

## Introduction

Bevacizumab is a monoclonal antibody targeting vascular endothelial growth factor (VEGF)-A and is widely used in the treatment of various solid malignancies, including ovarian cancer. By suppressing angiogenesis and vascular permeability, bevacizumab enhances the efficacy of chemotherapy and has become an essential component of oncologic regimens worldwide (1–3). However, inhibition of VEGF signaling also disrupts endothelial homeostasis, leading to a spectrum of adverse events. Among renal toxicities, proteinuria and hypertension are the most characteristic, occurring in up to 30% of patients, whereas nephrotic-range proteinuria is reported in 1 to 3% (4).

Although bevacizumab-associated proteinuria is well recognized, the underlying kidney pathology has been reported only infrequently. Previous case reports and small series have described thrombotic microangiopathy (TMA)-like lesions, but most lacked longitudinal detail or involved isolated cases across heterogeneous malignancies (5). In particular, data describing the temporal relationship between bevacizumab exposure, onset of proteinuria, and resolution after drug discontinuation remain limited.

Here, we present three ovarian cancer patients who developed proteinuria during long-term bevacizumab therapy, including one with biopsy-proven glomerular microangiopathy. Our series illustrates the consistent timing of onset, the reversibility of proteinuria after drug discontinuation, and the feasibility of conservative monitoring in patients with preserved kidney function. These findings provide practical insights for the clinical management of bevacizumab-associated kidney toxicity in onconephrology practice.

## Case presentation

### Case 1

**Figure 1:**
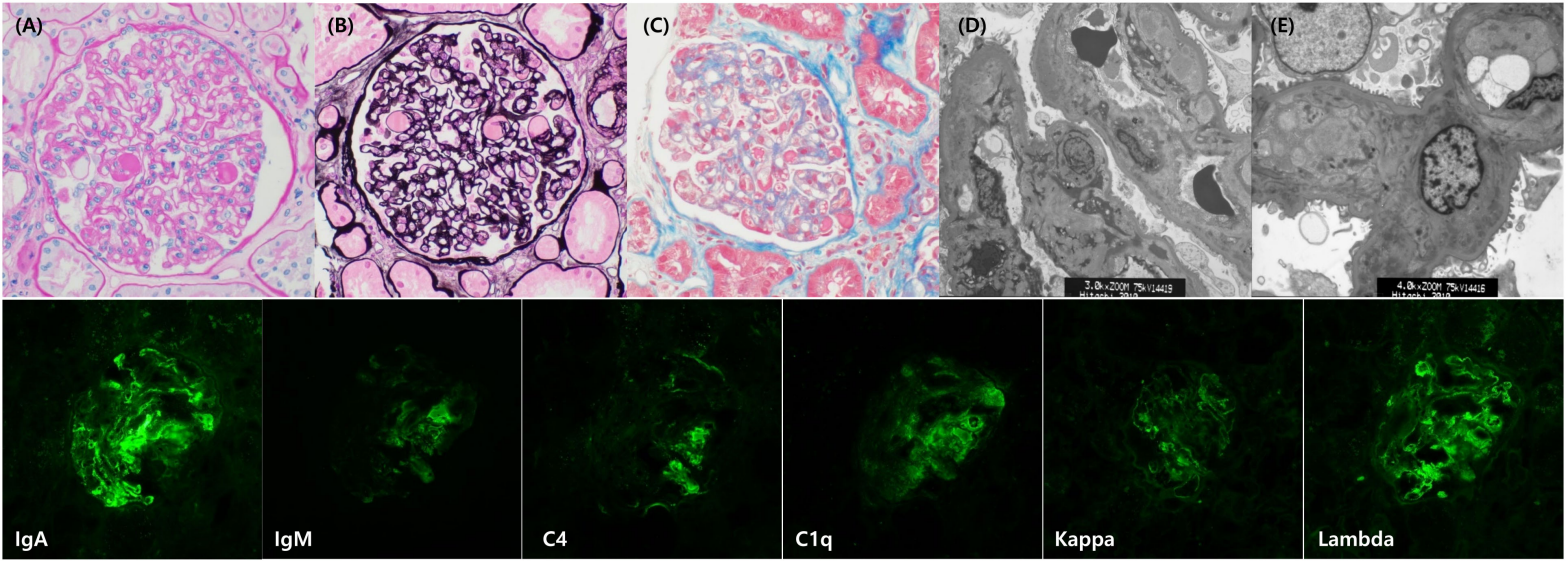
Kidney biopsy findings in a patient with bevacizumab-induced proteinuria. Light microscopy shows glomeruli with mesangial expansion and double-contour formation of the glomerular basement membrane **(A**, PAS; **B**, PASM; **C**, Masson’s trichrome**)**. Electron microscopy demonstrates subendothelial widening and electron-dense deposits with duplication of the basement membrane, consistent with glomerular microangiopathy **(D**, ×3,000; **E**, ×4,000**)**. Immunofluorescence reveals granular mesangial and capillary wall deposition of IgA, C4, C1q, and both κ and λ light chains, with weaker IgM staining, supporting immune complex–mediated injury.

A 56-year-old woman with stage IIIC serous ovarian carcinoma underwent optimal cytoreductive surgery followed by six cycles of paclitaxel–carboplatin, achieving remission. She had no history of diabetes mellitus, chronic kidney disease (CKD), or proteinuria prior to bevacizumab exposure. Hypertension was diagnosed approximately one year after initiation of bevacizumab and was well controlled thereafter. At first recurrence, she received six cycles of bevacizumab plus paclitaxel–carboplatin, then continued bevacizumab maintenance for a total of 37 months. Proteinuria was first detected during the maintenance phase at 12 months, with dipstick 1+ and urine protein-creatinine ratio (uPCR) 1.7 g/gCr, and progressed gradually to >4 g/gCr by month 24. Laboratory evaluation revealed no evidence of systemic TMA or secondary glomerular disease, with normal hemoglobin and platelet counts, normal serum albumin levels, absence of schistocytes on peripheral blood smear, normal complement levels (C3 and C4), negative antineutrophil cytoplasmic antibodies, and unremarkable coagulation parameters. Given the progressive proteinuria despite preserved kidney function, a kidney biopsy was performed, which demonstrated double-contour formation of the glomerular basement membrane and mesangial/subendothelial electron-dense deposits, consistent with glomerular microangiopathy (**Figure 1**). Kidney function was preserved, with estimated glomerular filtration rate (eGFR) consistently above 90 mL/min/1.73 m^2^. Bevacizumab was discontinued at month 37 due to disease progression, after which proteinuria rapidly declined and completely resolved within four months (**Table 1**).

**Table 1:** Clinical features and outcomes of the three patients with bevacizumab-associated proteinuria.

Case	Age/Sex	Cancer type	Bevacizumab exposure	Onset of proteinuria	Peak proteinuria (uPCR)	Peak serum creatinine	Biopsy findings	Outcome
1	56F	Serous carcinoma	37 months	12 months after initiation	4.6 g/gCr	0.83 mg/dL	Glomerular microangiopathy (TMA-like lesions)	Complete remission after 3 month of discontinuation
2	63F	Serous carcinoma	18 months	6 months after initiation	8.4 g/gCr	0.91 mg/dL	Not performed	Complete remission after 3 month of discontinuation
3	70F	Clear cell carcinoma	12 months	6 months after initiation	2.1 g/gCr	0.70 mg/dL	Not performed	Partial remission after 3 month of discontinuation

uPCR, urine protein-to-creatinine ratio; TMA, thrombotic microangiopathy.

### Case 2

A 63-year-old woman with serous ovarian carcinoma underwent staging surgery and six cycles of paclitaxel–carboplatin, achieving remission. She had no prior history of diabetes mellitus, CKD, or proteinuria. At recurrence, she received six cycles of bevacizumab plus paclitaxel–carboplatin, followed by bevacizumab maintenance therapy. Proteinuria was developed approximately two months after initiation of maintenance therapy and showed a progressive increase over time, reaching the nephrotic range several months later and peaking at a uPCR of 8.4 g/gCr. There were no clinical or laboratory features suggestive of systemic TMA, with preserved hemoglobin and platelet counts and no evidence of hypoalbuminemia. Kidney biopsy was not performed. Bevacizumab was discontinued after a total exposure of 18 months due to ovarian cancer progression. Proteinuria decreased within one month and fully resolved within six months of discontinuation (**Table 1**). Kidney function was preserved throughout treatment.

### Case 3

A 70-year-old woman with ovarian clear cell carcinoma underwent cytoreductive surgery followed by six cycles of paclitaxel–carboplatin. She had a history of hypertension diagnosed several years prior to bevacizumab exposure, which was well controlled, with no pre-existing diabetes mellitus, chronic kidney disease, or proteinuria. At disease recurrence, she received resection of peritoneal metastases and subsequently six cycles of bevacizumab plus paclitaxel–carboplatin, followed by maintenance bevacizumab. Proteinuria emerged during the maintenance phase, approximately 8 months after initiation of maintenance therapy. Systemic features of TMA were not observed, including anemia, thrombocytopenia, or hypoalbuminemia. Bevacizumab was discontinued due to progression of underlying malignancy, after which proteinuria gradually improved from subnephrotic-range levels to dipstick 1 plus within three months of discontinuation. Kidney function has remained well preserved with no additional kidney complications to date (**Table 1**).

## Discussion

Bevacizumab is a widely used anti-VEGF therapy that has become integral to modern oncologic practice, yet its renal toxicities remain incompletely understood. Proteinuria is the most frequent kidney complication, affecting up to one third of treated patients, but the pathologic spectrum and longitudinal outcomes are less well defined. Our series of three ovarian cancer patients provides practical insights into the clinical course of bevacizumab-induced proteinuria, including biopsy-proven glomerular microangiopathy and divergent trajectories after drug discontinuation.

From these cases, several key patterns emerge, particularly regarding the timing of onset, reversibility, and preservation of kidney function. Proteinuria appeared after 6–12 months of bevacizumab exposure, consistent with prior reports of onset within the first year (4). In all three patients, kidney function well preserved throughout treatment despite the development of significant proteinuria, in line with prior observations that bevacizumab-induced proteinuria rarely progresses to chronic kidney disease (6). Importantly, in both patients who discontinued bevacizumab, proteinuria resolved within 3–6 months, supporting the reversibility of this adverse effect. Given that bevacizumab-associated kidney injury, particularly kidney-limited TMA, may present with non-albuminuric or mixed proteinuria, combined assessment using urine dipstick testing and uPCR may be more informative than albumin-based measures alone in clinical practice.

Histologically, the observed double-contour basement membranes and subendothelial deposits are characteristic features of thrombotic microangiopathy (TMA). VEGF inhibition disrupts endothelial homeostasis and podocyte–endothelial crosstalk, leading to microangiopathic injury distinct from classic hemolytic TMA (7). Rare cases of bevacizumab-induced systemic TMA requiring eculizumab therapy have been reported (8). In contrast, our patients demonstrated kidney-limited TMA without systemic features such as hemolysis or thrombocytopenia. While close follow-up is warranted to monitor kidney function, current evidence suggests that kidney-limited TMA is generally manageable with careful monitoring rather than systemic therapy.

From a clinical standpoint, the key challenge lies in balancing oncologic benefit against renal risk. Continuing bevacizumab may be reasonable in patients with preserved kidney function when therapeutic efficacy is critical, while discontinuation often results in resolution of proteinuria and should be considered when toxicity or disease progression predominates. Although our observations are limited by the small number of cases, a single biopsy, and incomplete follow-up in one patient, as well as follow-up assessment of proteinuria primarily using urine dipstick rather than quantitative uPCR, they underscore the importance of shared decision-making between oncologists and nephrologists.

In conclusion, bevacizumab-induced proteinuria typically emerges within the first year of therapy and often resolves after discontinuation. Our series, which includes a biopsy-proven case of kidney-limited TMA, adds longitudinal evidence for the reversibility of bevacizumab-related nephrotoxicity.

## Data Availability

The raw data supporting the conclusions of this article will be made available by the authors, without undue reservation.
